# The use of High-Flow Nasal Oxygen Therapy in 4 dogs undergoing bronchoscopy

**DOI:** 10.3389/fvets.2023.1088103

**Published:** 2023-03-30

**Authors:** Maria Rosa de Jaureguizar Tesas, Hannah Matson, Simon Tappin, Emily Thomas

**Affiliations:** ^1^Department of Emergency and Critical Care, Veterinary Teaching Hospital, Georgia University (UGA), Athens, GA, United States; ^2^Department of Emergency and Critical Care, Royal Veterinary College, London, United Kingdom; ^3^Department of Internal Medicine, Dick White Referrals (Part of Linnaeus Veterinary Limited), Newmarket, United Kingdom; ^4^Department of Emergency and Critical Care, Dick White Referrals (Part of Linnaeus Veterinary Limited), Newmarket, United Kingdom

**Keywords:** High-Flow Nasal Oxygen Therapy, bronchoscopy, dogs, hypoxemia, oxygen

## Abstract

**Introduction:**

High-Flow Nasal Oxygen Therapy is a method to deliver warmed, humidified air-oxygen blended at high flow rates to patients through a nasal cannula using a specialized, commercially available machine. This is a well-tolerated, safe and effective method for oxygen delivery to healthy and hypoxemic dogs. Patients undergoing bronchoscopic procedures frequently develop hypoxemia. Human trials have shown a reduction in incidents of hypoxemic events and higher pulse oximeter oxygen saturation during bronchoscopies in patients on High-Flow Nasal Oxygen.

**Materials and methods:**

This is a single-centre, prospective case series. All dogs weighing between 5 and 15 kg and undergoing bronchoscopy during the study period (03/07/2022-01/10/2022) were eligible.

**Results:**

Twelve patients were eligible for inclusion of which four were enrolled. No clinically significant complications related to the use of High-Flow Nasal Oxygen Therapy were recorded. Two of the patients were re-intubated post bronchoscopy due to clinician preference for recovery. One of the patients had a self-limiting period of severe hypoxemia with a pulse oximeter oxygen saturation of 84% for < 1 min during bronchoalveolar lavage, and whilst undergoing High-Flow Nasal Oxygen administration. Another patient had a self-limiting episode of mild hypoxemia (SpO_2_ of 94% lasting < 1 min) 5 min after completion of bronchoalveolar lavage.

**Conclusion:**

No clinically relevant complications relating to High-Flow Nasal Oxygen Therapy were recorded in this case series, although further studies are required to confirm this conclusion. This initial data suggests that the use of High-Flow Nasal Oxygen therapy during bronchoscopy is feasible and potentially safe, although it may not prevent hypoxemia in these patients. The use of High-Flow Nasal Oxygen Therapy during bronchoscopy in small patients carries multiple potential benefits and further studies to compare its efficacy against other traditional oxygen delivery systems are warranted in this patient population.

## 1. Introduction

High-Flow Nasal Oxygen Therapy (HFNOT) is an increasingly available method in veterinary medicine to deliver warmed, humidified, blended air-oxygen, at 95%-100% relative humidity and at high flow rates to patients through a nasal cannula using a specialized, commercially available machine (1, 2). The fraction of inspired oxygen (FiO_2_) can be independently adjusted on some HFNOT devices from 21 to 100% (1–3). HFNOT has shown to be a well-tolerated, safe and effective method for oxygen delivery to healthy and hypoxemic dogs (2–8).

In human and veterinary medicine, patients undergoing bronchoscopic procedures may develop hypoxemia. Bronchoalveolar lavage can be one of the most critical moments during the bronchoscopy, with the instillation of saline causing ventilation/perfusion (V/Q) mismatch and creation of venous admixture. This can be exacerbated by subsequent suction resulting in reduced pressures within the alveoli and potential alveolar collapse. Sedation and general anesthesia may reduce respiratory drive and lead to hypoventilation, and partial occlusion of the airway by the bronchoscope may exacerbate this (9). In the authors' experience, small breed dogs requiring extubation because the inner diameter of the endotracheal tube is too small for insertion of a standard bronchoscope may have an increased risk of developing hypoxemia. Oxygen is typically supplemented during bronchoscopy using conventional sources and flow rates. However, human trials have shown a reduction in the incidence of hypoxemic events and higher oxygen saturation (SpO_2_) during bronchoscopies in patients on HFNOT compared to patients receiving conventional oxygen therapy with nasal prongs or cannula (9–16).

To the authors' knowledge, the safety and feasibility for the use of HFNOT during bronchoscopic procedures in dogs have not been evaluated. The objective of the case series reported here was to evaluate the safety and feasibility of use of HFNOT in dogs undergoing bronchoscopy that required extubation for the procedure (i.e. small breeds).

## 2. Materials and methods

### 2.1. Study population and setting

This is a single-centre, prospective case series. All dogs weighing between 5 and 15 kg and undergoing bronchoscopy during the study period (March 7th 2022–October 1st 2022) were eligible for inclusion.

Exclusion criteria included: obstructive nasal disease, airway disruption (e.g., tracheal tear, tracheostomy tube), suspected or confirmed highly contagious disease (e.g., infectious tracheobronchitis, infection with multidrug resistant bacteria), pneumothorax (suspected by point of care ultrasound or confirmed with thoracic radiography), and clinician suspicion of increased intracranial pressure. Patients where endotracheal tube removal was not necessary for bronchoscopy were also excluded, as were those where the HFNOT unit was in use with another patient, or there was not a technician available to record the data and run the machine.

The study was approved by the ethical review committee used by our institution (ethics review number 3065 200106). The ethical review committee deemed it unnecessary to obtain written consent for participation from owners because oxygen delivery is regarded as a normal standard of care in these patients. HFNOT is increasingly being used in our institution for this purpose and therefore the study protocol was not deemed to differ from normally offered care.

### 2.2. Experimental procedure and data collection

Baseline patient information including age, gender, breed, weight, reason(s) for bronchoscopy, comorbidities and current medications were recorded. All dogs were anesthetized using an initial induction bolus of intravenous propofol (PropoFlo Plus 28, 200 mg/20 mL, Zoetis, United States) given to effect, followed by a continuous rate infusion of 0.1–0.4 mg/kg/minute. All patients were premedicated with different protocols according to clinician discretion. Those protocols were recorded. All dogs were intubated after anesthetic induction and received oxygen *via* endotracheal tube.

**Figure 1:**
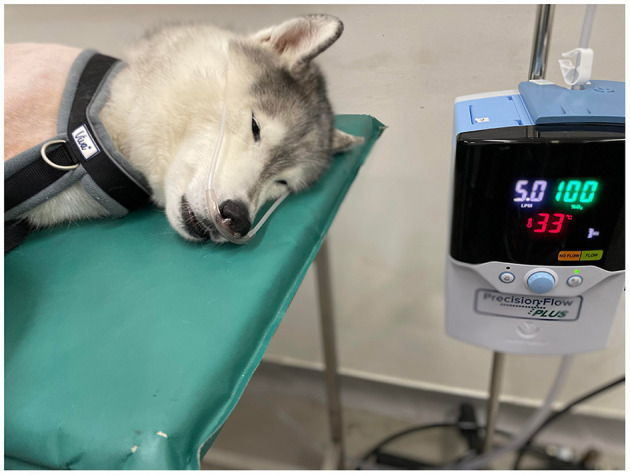
High-Flow Nasal Oxygen Machine (Precision Flow Hi-VNI, Vapotherm, United States) and positioning of the nasal prongs. This patient was not included in this case series.

Proprietary nasal prongs (High Velocity Nasal Insufflation [Hi-VNI] Cannula, Vapotherm, United States) were placed in the nares during general anesthesia immediately prior to endotracheal extubation before bronchoscopy (Figure 1). A prong size to occupy ~50% of the diameter of the nares was chosen, and the prong size recorded. Gas flows of 1 L/kg/min at a fraction of inspired oxygen (FiO_2_) 100% and a temperature of 33°C were delivered *via* the High-Flow Nasal Oxygen Machine (Precision Flow Hi-VNI, Vapotherm, United States) (Figure 1) after the patient was extubated immediately prior to bronchoscopy. The flow rate was rounded up or down from the nearest 0.5 L to a non-decimal value. No changes were made to the HFNOT settings during the procedure.

Pulse oximeter oxygen saturation (SpO_2_) was recorded every minute and heart rate, respiratory rate, temperature, systolic, diastolic and mean blood pressures were recorded every 5 min with a multiparameter monitor (UMEC12 Vet, Mindray animal care, China). Severe hypoxemia was defined as SpO_2_ below 90% (based on definitions in human studies) and mild hypoxemia as SpO_2_ below 95%. In the event of hypoxemia, treatment was based on clinician preference. Data was collected from the time of extubation and beginning of the bronchoscopy throughout the procedure until the nasal cannula was removed once the patient was fully recovered, or until the patient was re-intubated. The time between extubation and beginning of the bronchoscopy was negligible. The reason for any re-intubation was recorded. Complications potentially related to the use of HFNOT were monitored for and recorded. Complications were defined as respiratory (respiratory rate > 40 breaths per minute, or prolonged severe or mild hypoxemia for more than 5 min) or non-respiratory (intolerance to nasal prongs defined as patient rubbing face, onset of epistaxis, hyperthermia defined as rectal temperature above 39.1°C). If noted, respiratory complications were further investigated with thoracic radiographs or thoracic point of care ultrasound.

If the patient had a BAL during the procedure it was recorded. BAL was performed by instilling 3–5 mL aliquots of sterile saline through the wash channel of the wedged bronchoscope and collecting using no more than 10 cm of water suction into a wash trap. However, how much sterile saline was used for the specific patient and how much sample was retrieved was not recorded. Bronchoscopic findings and BAL cytology results were collected.

### 2.3. Statistical analysis

Statistical analysis was performed by use of standard statistical software (Microsoft Excel 14.5.4, Microsoft Corporation, 2011). Baseline patient information and monitoring data recorded during anesthesia were assessed for normality using a dispersion graph. All data were non-normally distributed and are presented as median (range).

## 3. Results

Twelve patients were eligible for inclusion of which four were enrolled. Eight patients were excluded, 2/8 because the HFNOT machine was in use with another patient and 6/8 because a technician was not available to run the machine during the procedure. One each of Shih Tzu (case 1 in Tables 1, 2), French Bulldog (case 2 in Tables 1, 2), Jack Russell Terrier (case 3 in Tables 1, 2) and small mixed breed (case 4 in Tables 1, 2) were included, of which 2/4 were male castrated (case 3 and 4), 1/4 was male intact (case 2) and 1/4 was female spayed (case 1). The median age was 11 years 3 months (1–14 years) and median weight was 9.35 kg (6.25–14.8 kg). Three of the patients enrolled had other comorbidities. Two of them had myxomatous mitral valve disease (MMVD), one at stage B1 (case 3) and the other at stage C (case 1) (17). The patient with MMVD stage C was on oral pimobendan, oral furosemide, oral amlodipine, oral benazepril, oral marbofloxacin and oral prednisolone prescribed by the referring veterinarian. One of the patients had brachycephalic obstructive airway syndrome (case 2). The reason for bronchoscopy in all four patients was further investigation of chronic cough. A BAL was performed in all four patients. Patient baseline characteristics are summarised in Table 1.

**Table 1:** Patient characteristics.

**Patient identification**	**Age**	**Gender**	**Breed**	**Weight**	**Comorbidities**
1	14 years	Female spayed	Shih Tzu	6.25 kg	Myxomatous Mitral Valve Disease Stage C
2	1 year	Male intact	French Bulldog	14.8 kg	Brachycephalic Obstructive Airway Syndrome
3	12 years 6 months	Male castrated	Jack Russell Terrier	8 kg	Myxomatous Mitral Valve Disease Stage B1
4	9 years 9 months	Male castrated	Mixed breed	10.7 kg	None

**Table 2:** Monitoring parameters and duration of bronchoscopy and HFNOT.

**Patient identification**	**1**	**2**	**3**	4^*^	**Median (range)**
Duration of bronchoscopy (minutes)	15	5	26	4	10 (4–26)
Duration of HFNOT (minutes)	25	5	26	13	19 (5–26)
Heart rate (bpm) (minimum–maximum)	82–127	80–96	85–97	90	90 (80–127)
Respiratory rate (bpm) (minimum–maximum)	10–12	16–20	8–12	15	11 (8–20)
Systolic arterial pressure (mmHg) (minimum–maximum)	94–118	114–130	99–103	125	103 (94–130)
Mean arterial pressure (mmHg) (minimum–maximum)	59–76	55–79	62–108	83	70 (59–108)
Diastolic arterial pressure (mmHg) (minimum–maximum)	42–88	49–54	51–102	69	57 (42–102)
Temperature (°C) (minimum–maximum)	33.4–36.1	35.9	36.3–36.7	37.9	36.3 (33.4–37.9)
SPO2 (%) (minimum–maximum)	84–100	98–100	94–100	96–99	99.5 (96–100)^**^
Size of proprietary nasal prongs	Pediatric	Pediatric/adult	Pediatric	Pediatric/adult	

* Duration of bronchoscopy in Patient number 4 was too short for multiple readings of most parameters, other than SPO_2_.

** Initial values only.

The patient with MMVD stage C was premedicated with intravenous butorphanol 0.2 mg/kg (Torbugesic 10 mg/mL, Zoetis, Spain) and intravenous acepromazine 10 mcg/kg (Acecare 2 mg/mL, AnimalCare, Netherlands), the other three patients received intravenous butorphanol 0.2-0.4 mg/kg (Torbugesic 10mg/mL, Zoetis, Spain) and intravenous dexmedetomidine 1–2 mcg/kg (Dexdomitor 0.5 mg/mL, Zoetis, Finland). Anesthesia of every patient was induced with intravenous propofol (PropoFlo Plus 28, 10 mg/mL, Zoetis, United States) and patients were intubated prior to bronchoscopy.

Proprietary nasal prongs (High Velocity Nasal Insufflation [Hi-VNI] Cannula, Vapotherm) were placed in the nares after induction of anaesthesia and endotracheal extubation. The cannula tubing was positioned to pass under the patients' ears on both sides and was gently tightened until a snug fit was achieved. In two of the patients (cases 1 and 3) the pediatric size was used, and in two (cases 2 and 4) the pediatric/adult size was used. The median bronchoscopy duration was 10 min (4–26 min).

All the patients had a pulse oximeter placed on the tongue. The initial SPO_2_, immediately after starting HFNOT, ranged between 96 and 100%, with a median of 99.5%. The lowest SPO_2_ was 84% in the patient with MMVD stage C disease. This patient had a single, self-limiting period of severe hypoxemia with a SpO_2_ of 84% lasting for < 1 min and occurring 5 min into bronchoscopy and during the BAL procedure. There was no concurrent change in heart rate or mucous membrane colour. Another patient (case 3) had a self-limiting episode of mild hypoxemia with a SpO_2_ of 94% lasting < 1 min, over 20 min into the bronchoscopy and 5 min after the end of the BAL.

The median heart rate was 90 beats per minute (80–127 beats per minute) and median respiratory rate was 11 breaths per minute (8–20 breaths per minute). The patients' systolic arterial pressure ranged from 94 to 130 mmHg, mean arterial pressure between 59 and 108 mmHg and diastolic arterial pressure between 42 and 102 mmHg. The median pressures were 103, 70, and 57 mmHg for systolic, mean and diastolic, respectively. The median temperature was 36.3°C (33.4 to 37.9°C). Three of the patients (cases 1, 2 and 3) had a temperature below 37.5°C prior to and during the procedure with an improvement in two patients (cases 1 and 3) on heat support.

Two of the patients (cases 2 and 3) were re-intubated post bronchoscopy due to clinician preference for recovery. Re-intubation occurred immediately after the procedure with negligible time on HFNOT between the end of the procedure and re-intubation. Two of the patients (cases 1 and 4) were not re-intubated after bronchoscopy was finished and data was recorded until the patient was awake and fully recovered. Case 1 remained on HFNOT for a total of 25 min, which included an additional 10 min from the end of bronchoscopy, and case 4 remained on HFNOT for 13 min, including 9 min from the end of bronchoscopy. These patients had no recorded hypoxemia during their recovery time.

Patient monitoring values during bronchoscopy are summarised in Table 2. No complications related to the use of HFNOT were recorded.

All patients had abnormal bronchoscopic findings and 3/4 (75%) of patients had abnormal BAL results. Bronchoscopy findings and BAL results are summarised in Table 3.

**Table 3:** Bronchoscopy and BAL results.

**Patient identification**	**Bronchoscopy findings**	**Bronchoalveolar lavage results**
1	Moderate bronchiectasia, mucus plugging, dynamic bronchial collapse	Eosinophilic inflammation, neutrophilic inflammation, goblet cell hyperplasia
2	Mild bronchiectasia	Bacterial infection
3	Mild bronchiectasia, mucus plugging, tracheal collapse grade 1	Normal
4	Mild bronchiectasia, mucus plugging, tracheal collapse grade 2, dynamic bronchial collapse	Eosinophilic inflammation and neutrophilic inflammation

## 4. Discussion

HFNOT is widely used to treat respiratory distress and hypoxemia in people, and increasingly used in veterinary medicine. HFNOT delivers warmed, humidified blended medical air with oxygen at high flow rates to patients through a nasal cannula. There are several commercially available HFNOT delivery units available, which can deliver oxygen flow rates from 1 to 60 L/min. The cartridges used in this case series allowed flow rates from 5 to 40 L/min, hence the lower weight limit of 5 kg for inclusion. The fraction of inspired oxygen (FiO_2_) can be independently adjusted on some HFNOT devices from 21 to 100% although the FiO_2_ delivered to the alveoli is lower than the HFNOT machine setting (1, 3, 18). One experimental model showed that a machine set to deliver 100% FiO_2_ only achieved a true FiO_2_ delivery of 78.67 and 76.67% at 300 and 500 mL tidal volumes, respectively (18). However, another study reported higher FiO_2_ values ranging from 86 to 97% at HFNOT settings of 100% FiO_2_ with flow rates ≥1 L/kg/min (6). The physiological effects of HFNOT are thought to include improvement of mucociliary function, enhanced secretion clearance and reduction in ventilation/perfusion mismatch (V/Q mismatch) (1, 19). The use of warm, humidified gas reduces bronchoconstriction in people (20). The flow-dependent continuous positive airway pressure (CPAP) may reduce atelectasia, improve functional residual capacity and prevent lung de-recruitment. HFNOT decreases anatomical dead space through nasopharyngeal “purging” of CO_2_ and net positive pharyngeal pressure, leading to reduction of CO_2_ rebreathing and thus improving ventilation and avoiding increased diaphragm activation during bronchoscopy and BAL (1, 13, 21–23). The reduction of resistance in the upper airways improves respiratory dynamics and reduces the minute ventilation necessary to provide appropriate alveolar ventilation.

In human patients in respiratory failure, HFNOT has been shown to increase the degree of comfort while reducing both respiratory rate and the severity of dyspnea (24). While on HFNOT, in comparison with conventional oxygen therapy, there is less disruption of eating and drinking in people, although this may in part be due to the fact that patients on HFNOT do not have to wear a tight-fitting mask (24). Those findings might be the result of the heating and humidification of inspired gases, as cold dry gas is associated with decreased compliance, conductance and bronchoconstriction (25, 26).

Multiple veterinary studies have assessed the safety and efficacy of HFNOT in healthy dogs and in dogs with respiratory disease. One study compared PaO_2_ in sedated, healthy, non-brachycephalic dogs receiving conventional oxygen therapy at 100 mL/kg/min *via* nasal canulae, followed by HFNOT at 20 and 30L/min. PaO_2_ was significantly higher during HFNOT (at both 20 and 30 L/min) than during conventional oxygen therapy (7). A retrospective study describes six hypoxemic dogs that failed to respond to conventional oxygen therapy, and were then treated with HFNOT. The PaO_2_ was significantly higher after HFNOT was started and four of the patients had resolution of the hypoxemia with three surviving to discharge. One patient required sedation but HFNOT was otherwise well tolerated (5). Successful use of HFNOT after extubation has been reported in a dog with severe bronchoconstriction, suspected to be caused by an anaphylactic reaction (27). Several prospective trials have enrolled dogs with hypoxemic respiratory failure that did not respond to conventional oxygen therapy (2, 28). Overall, these trials showed a significant improvement in PaO_2_ after institution of HFNOT, and therapy was typically well tolerated (28). Similarly, a study investigating recovery from general anesthesia in dogs with obstructive upper airway disease showed an improved dyspnea score in patients treated with HFNOT, and the device was well tolerated (29).

The HFNOT gas flow rates chosen in this case series were broadly based on settings described in the veterinary studies above and in human guidelines. Lower flow rates are better tolerated in people, but nonetheless a flow rate of 60 L/min is recommended for rapid relief of dyspnea in people, approximating to our chosen flow rate of 1 L/kg/min (30). HFNOT is generally well tolerated in conscious dogs at flow rates up to 2 L/kg/min but one study showed that at flow rates of >2 L/kg/min patients showed reduced tolerance to the device, becoming agitated and pawing their face (6). The gas temperature of 33 °C was chosen based on the temperatures typically used in conscious patients at our institution. Studies have shown that at the same oxygen rate, people seemed to be more comfortable at lower temperatures (31°C instead of 37°C) (26). However, in dogs recovering from general anesthesia, there was no difference in tolerance between lower vs. higher temperatures (4). Higher gas temperatures could therefore be considered in future studies of HFNOT in anesthetized dogs.

Bronchoscopy and BAL are commonly performed in dogs and cats with respiratory disease. These procedures are not innocuous and can be associated with complications including hypoxemia, atelectasis, bronchospasm, bradycardia, hypercapnia and pneumothorax (31–33). Hypoxemia can be exacerbated in small breed dogs where the endotracheal tube internal diameter is too small (< 7.5 mm) to fit a standard bronchoscope, thus extubation is required. The upper size limit in our population was chosen to reflect these patients. Mild to moderate hypoxemia during bronchoscopy has been reported in up to 18% dogs undergoing computed tomography and BAL (34). Rarely, patients may deteriorate significantly, and both death and requirement for mechanical ventilation have been reported, although this is in patients with significant underlying disease (35, 36). The British Thoracic Society guidelines for diagnostic flexible bronchoscopy in adults recommend oxygen supplementation when there is significant desaturation (drop in SPO_2_ of more than 4% or SPO_2_ below 90%) prolonged over 1 min (36). Reports in human medicine showed that oxygen supplementation is required in 24% of patients undergoing bronchoscopy (37). Human research has shown HFNOT to be superior to conventional oxygen therapy by reducing the incidence of hypoxemia (< 90%) during bronchoscopy (10–16).

In this case series, one of the patients became severely hypoxemic during its BAL and whilst on HFNOT, although this was only for a brief period and was self-limiting. Thus, HFNOT did not prevent hypoxemia in all patients. Since the soft palate is displaced by the bronchoscope during bronchoscopy and may obstruct the passage of oxygen supplemented *via* nasal prongs, this is perhaps not surprising. The affected patient did not show hemodynamic changes related to hypoxemia and, as discussed below, pulse oximetry can be insensitive for measurement of hypoxemia. We did not perform any further diagnostic testing (e.g., arterial blood has analysis) to confirm hypoxemia or investigate its physiological cause in this patient. However, given that the hypoxemia occurred during BAL and was self-limiting it was deemed most likely secondary to the procedure (airway obstruction and/or bronchoconstriction). In future studies assessing the effect of HFNOT during bronchoscopy, in the event of hypoxemia, removal of the HFNOT device to see whether the hypoxemia resolves could be considered as a simple method to help differentiate whether the device itself is contributing to the hypoxemia. Human research has shown that non-invasive ventilation providing continuous positive airway pressure (CPAP) using a mask is superior to HFNOT for providing oxygenation before, during and after bronchoscopy in patients with moderate to severe hypoxemia (38). However, the mask required is not always well tolerated by the patient and it is difficult to access the airway with the scope (14). In addition, none of the patients in this case series were initially hypoxemic. Non-invasive ventilation provision of CPAP using a mask or pediatric helmet has been investigated in veterinary medicine, although not during bronchoscopy (39–44). However, similar to humans, this may be poorly tolerated and/or impede airway access with the scope in this situation. Similarly, intubation and intermittent positive pressure ventilation could be used to treat hypoxemia but would preclude concurrent bronchoscopy and BAL in these small patients owing to the size of the endotracheal tube. However, comparative prospective studies would be necessary to investigate the superiority of HFNOT to conventional oxygen delivery systems before, during and after bronchoscopy in veterinary patients. Similarly, the HFNOT gas flow rates and temperature settings chosen for this case series were broadly based on settings that had been previously described in veterinary studies (2, 4–8). However, to date, HFNOT has predominantly been investigated in conscious or sedated dogs. Thus, future studies comparing the tolerance and efficacy of a variety of flow rates and temperature settings are warranted in this anesthetized population where intubation is not possible.

Complications of HFNOT appear to be minimal in dogs, although data on the use of this treatment modality are still limited. Most of the complications reported to date in veterinary patients are minor, such as intolerance of nasal prongs as described above (2, 4–8) and aerophagia (6, 7, 29). More serious complications such as development of pneumothoraces have been described in children, but are relatively uncommon (45). No clinically significant complications derived from the use of HFNOT were reported in the dogs undergoing bronchoscopy in this case series. However, we assessed for respiratory complications on the basis of clinical examination and since no dogs met our criteria for a clinical suspicion of a complication, thoracic radiography or point of care ultrasound was not performed in any dog. Thus, we cannot rule out subclinical complications such as low volume pneumothorax.

There are further limitations to this case series. The sample size is small, but we aimed only to assess safety and feasibility: further prospective studies are needed to compare the efficacy of HFNOT vs. conventional oxygen supplementation and/or non-invasive CPAP provision. Hypoxemia was assessed using pulse oximetry, chosen as a pragmatic method to allow constant monitoring of oxygenation in patients where dynamic changes were expected. However, although SPO_2_ relates to the partial pressure of oxygen (PaO_2_) using the oxyhemoglobin dissociation curve, it can be insensitive to detect hypoxemia in dogs (46). Different temperatures may affect the haemoglobin affinity for oxygen. Therefore, hypothermia may shift the oxygen dissociation curve to the left, increasing haemoglobin's affinity for oxygen. Multiple factors may affect the accuracy of pulse oximetry in dogs, including vasoconstriction and resultant reduced perfusion secondary to hypovolaemia, hypotension or hypothermia, and the anatomical monitoring site, including haired, thick, or pigmented areas (47). Based on blood pressure measurement, our patient population was assumed to be well perfused, and pulse oximetry site was standardized to the tongue to avoid variation. Nonetheless, arterial blood sampling and measurement of PaO_2_ would have been gold standard. Blood gas analysis would, additionally, have enabled measurement of PaCO_2_ and assessment of the contribution of hypercapnia to hypoxemia. Two patients in this case series were reintubated for recovery, based on clinician preference. Data were not collected regarding the reasoning behind this choice. However, since reintubation may be indicative of post-procedural failure this could be a useful additional outcome measure to assess in future comparative studies.

In conclusion, no dogs showed clinically relevant complications. This initial data suggests that the use of HFNOT during bronchoscopy in dogs is feasible and appeared to be safe in the limited number of patients in this case series. However, transient hypoxemia was observed in one patient thus it may not prevent hypoxemia. The benefits of using High-Flow Nasal Oxygen Therapy during bronchoscopy have been well described in people, and, given the difficulties of maintaining oxygenation during bronchoscopy in small patients that require extubation, its use in this patient population warrants further evaluation.

## Data availability statement

The raw data supporting the conclusions of this article will be made available by the authors, without undue reservation.

## Ethics statement

The animal study was reviewed and approved by Chair, Ethics Clinical Review Panel, School of Veterinary Medicine, University of Nottingham. The Ethical Review Panel deemed it unnecessary to obtain written consent for participation from owners because oxygen delivery is regarded as a normal standard of care in these patients. High flow nasal oxygen therapy is increasingly being used in our institution for this purpose and therefore the study protocol was not deemed to differ from normally offered care.

## Author contributions

ET, ST, and HM contributed to design the study. MJ and ET organized the database. MJ wrote the first draft of the manuscript. MJ, ST, HM, and ET wrote sections of the manuscript. All authors contributed to manuscript revision, read, and approved the submitted version.
